# Comparison of an Ultrasound-Assisted Aqueous Two-Phase System Extraction of Anthocyanins from Pomegranate Pomaces by Utilizing the Artificial Neural Network–Genetic Algorithm and Response Surface Methodology Models

**DOI:** 10.3390/foods13020199

**Published:** 2024-01-08

**Authors:** Qisheng Yue, Jun Tian, Ling Dong, Linyan Zhou

**Affiliations:** 1 Faculty of Food Science and Engineering, Kunming University of Science and Technology, Kunming 650500, China; yueqisheng0202@163.com (Q.Y.); tj7920921@163.com (J.T.); 2 Yunnan Engineering Research Center for Fruit & Vegetable Products, Kunming 650500, China; 3 Yunnan Key Laboratory for Food Advanced Manufacturing, Kunming 650500, China; 4 International Green Food Processing Research and Development Center of Kunming City, Kunming 650500, China; 5 Faculty of Information Engineering and Automation, Kunming University of Science and Technology, Kunming 650500, China

**Keywords:** by-product, cyanidin-3-O-glucoside, ultrasound-assisted aqueous two-phase extraction, genetic algorithm, pomegranate pomace

## Abstract

As a by-product of pomegranate processing, the recycling and reuse of pomegranate pomaces (PPs) were crucial to environmentally sustainable development. Ultrasound-assisted aqueous two-phase extraction (UA-ATPE) was applied to extract the anthocyanins (ACNs) from PPs in this study, and the central composite design response surface methodology (CCD-RSM) and artificial neural network–genetic algorithm (ANN-GA) models were utilized to optimize the extraction parameters and achieve the best yield. The results indicated that the ANN-GA model built for the ACN yield had a greater degree of fit and accuracy than the RSM model. The ideal model process parameters were optimized to have a liquid–solid ratio of 49.0 mL/g, an ethanol concentration of 28 g/100 g, an ultrasonic time of 27 min, and an ultrasonic power of 330 W, with a maximum value of 86.98% for the anticipated ACN yield. The experimental maximum value was 87.82%, which was within the 95% confidence interval. A total of six ACNs from PPs were identified by utilizing UHPLC-ESI-HRMS/MS, with the maximum content of cyanidin-3-O-glucoside being 57.01 ± 1.36 mg/g DW. Therefore, this study has positive significance for exploring the potential value of more by-products and obtaining good ecological and economic benefits in the future.

## 1. Introduction

Pomegranate (*Punica granatum* L.) is regarded as a ‘Superfruit’ in the global functional food industry [1,2], leading to dramatic increases in demand for pomegranate fruit and its products in world trade, such as pomegranate juice, vinegar, wine, powder, and so on [3]. Thus, considerable quantities of pomegranate pomaces (PPs) are produced during pomegranate juicing, and the PP mainly consists of an internal membrane and aril fragments when juicing after peeling, which account for around 10% of the whole pomegranate [4]. High levels of bioactive substances, including anthocyanins (ACNs), flavonoids, tannins, and other phytonutrients, are still represented in these pomaces.

ACNs are water-soluble pigments, and more than 90% of ACN are found in all organs of the plant, imparting various hues of red, purple, and blue [5]. ACNs are commonly derived from the six most common anthocyanidins: petunidin (Pt), malvidin (Mv), peonidin (Pn), pelargonidin (Pg), cyanidin (Cy), and delphinidin (Dp) [6], which combine with sugar in glycosidic bonds. Since the structure of an ACN is relatively unstable, it is vulnerable to irreversible degradation due to changes in external environmental conditions, including light, temperature, pH, and oxygen [7]. ACNs have the potential to serve as an alternative to unnatural colorants because of their bright colors and water-soluble properties and outstanding achievements in the field of health promotion and multipurpose medicinal values, such as their anti-tumor, anti-hyperglycemia, and antioxidant properties; they also the risk of cardiovascular diseases [8]. However, the PP after juice processing was normally discarded, which results in environmental pollution and economic loss. With the development of the pomegranate industry, its by-products dramatically increase, and the problem becomes increasingly prominent. However, little research has focused on the utilization of the by-products of pomegranate, especially using green and sustainable methods. As a result, it was important to build an environmentally friendly and sustainable technique for extracting biologically active compounds. The objective of this study was to extract ACNs from PPs in a green and efficient technique.

The aqueous two-phase system (ATPS) consists of two non-compatible ingredients, which are generally polymers and salts, and the phase separation of the ATPS occurs when the proportion of the mixture exceeds the critical level [5]. The utilization of aqueous two-phase extraction (ATPE) has been significantly extended in recent years. For example, the utilization of the ATPE technique for the isolation of bioactive constituents was achievable due to its benefits such as a minimal impact on the biologically active compounds, convenient separation, reduced viscosity, and efficient solvent reuse [9,10,11]. Ultrasound generates mechanical, cavitation, and thermal effects in the extraction solvent system, resulting in an increase in the solvent into the target substance matrix and an acceleration of the release of the target substances into the extraction solvent system [12]. On the one hand, ultrasound also can disintegrate the participants into smaller pieces, thereby increasing the surface area available for extraction, promoting boundary layer renewal, and enhancing mass transfer between solvents through solvent diffusion and penetration. Thus, ultrasound-assisted extraction (UAE) can achieve the same extraction effect in a shorter time compared to conventional extraction [11].

In recent years, the combined applications of different approaches were popular for the extraction of bioactive substances, as they significantly improved the extraction yield of target compounds. The combination of ultrasound and ATPE, namely ultrasound-assisted aqueous two-phase extraction (UA-ATPE), may not only improve the extraction efficiency for biological activity substances but also enhance the target chemicals’ purity due to the selective migration of bioactive compounds to the bottom phase; meanwhile, the majority of impurities were transferred to the top phase. Previous studies have reported that UA-ATPE could be used for the extraction of phytoflavonoids and polysaccharides, and its extraction efficiencies were generally better than the conventional method [13]. Zhang et al. [11] compared the extraction difference of heat water extraction (HWE), UAE, and UA-ATPE applied for polysaccharides from *Lilium davidiivar. unicolor* Sali, and the yields of HWE, UAE, and UA-ATPE were 7.62, 12.86, and 36.58%, respectively; meanwhile, the polysaccharides extracted by UA-ATPE had the highest purity of 84.71%. Zhu et al. [13] compared the extraction efficiencies of UA-ATPE and ATPE applied for the flavonoids from jujube peels, and the optimized UA-ATPE and ATPE yields were 7.14 ± 0.25 and 4.87 ± 0.13 mg/g, respectively. Moreover, the microwave-assisted aqueous two-phase extraction (MA-ATPE) was successfully applied for the extraction and preliminary purification of ACNs from *Rosa pimpinellifolia* L. fruits (RPF) by Odabaş et al. [14], and results showed that the yield of ACNs from the optimized MA-ATPE extract was lower than the value of microwave-assisted extraction (MAE) alone, but the purity of ACNs was nearly 1.65-fold higher than MAE (3.61 ± 0.14%). However, for all we know, there was no study conducted regarding the application of UA-ATPE for the extraction of an ACN.

During the extraction process, the objective is first to determine the parameters of optimal extraction required to achieve better yields, and second to improve extraction efficiency, reduce unnecessary consumption, achieve higher purity of target substances, etc. The artificial neural network (ANN) and central composite design response surface methodology (CCD-RSM) are commonly used for modeling the extract process to forecast the yield of the desired compounds. The CCD-RSM was considered to be a conventional statistical technique that has been widely utilized to formulate experiments, establish models, and assess the influence of different processing parameters on extraction efficiency [15]. The ANN is a machine learning method inspired by human neurons, which can model intricate biological processes and highly non-linear outcomes effectively. It offers the benefit of solving complex problems unmanageable by artificial or statistical approaches [9,16]. The ANN has been found to be more effective for prediction and optimization than traditional regression models in numerous studies [11,12]. The genetic algorithm (GA) is a population optimization technique that uses natural selection theory and genetic principles to achieve random, adaptive, and global optimization [17]. The GA was a search method that has been shown to be an effective methodology for tackling numerous optimization problems because it mimics the adaptation process of real biological systems [18]. The integration of the ANN and GA methods was aimed at leveraging their respective strengths to tackle complex non-linear relationship modeling. The application of the ANN bonding with the GA (ANN-GA) for the optimization of the extraction process can help discover the most optimal conditions with less effort. Moreover, the GA added benefits of rectifying imperfections in optimized neural networks, circumventing the pitfalls of local extreme values, and achieving superior convergence and adaptability [19]. Recently, some studies have reported that the ANN-GA could be used to optimize the extraction parameters for target compounds from food raw materials or by-products, including ellagitannins from black raspberry seeds [20], punicalagin from pomegranates [21], and polyphenols from dragon fruit peels [22]. Those studies showed that the profound integration of the ANN and GA significantly enhanced the efficiency of identifying optimal extraction parameters during the extraction process, and generally yielded more accurate prediction results compared to traditional RSM. The utilization of the ANN-GA has emerged as a trending approach for modeling and optimization in the food processing industry.

In this study, in order to optimize the extraction process of ACNs from PPs, we first built a UA-ATPS for the extraction of the ACN from the PP by demonstrating the phase diagrams of ethanol and ammonium sulfate ratios, and then compared the optimization results of the UA-ATPE process by both CCD-RSM and the ANN-GA, including ACN yield, antioxidant activity, and monomeric ACN content. Finally, the extracted ACN was identified and quantified using ultra-high-performance liquid chromatography coupled to electrospray ionization high-resolution mass spectrometry (UHPLC-ESI-HRMS/MS). The target of this research was to establish a theoretical basis for the future application of UA-ATPE to extract ACNs from by-products of pomegranate and optimize the extraction procedure by utilizing CCD-RSM and an ANN-GA. In this work, an efficient and ecologically acceptable technique was developed for the extraction of the ACN from the PP, which was very promising for reducing by-product waste and increasing the value of pomegranate by-products.

## 2. Materials and Methods

### 2.1. Outline

**Figure 1:**
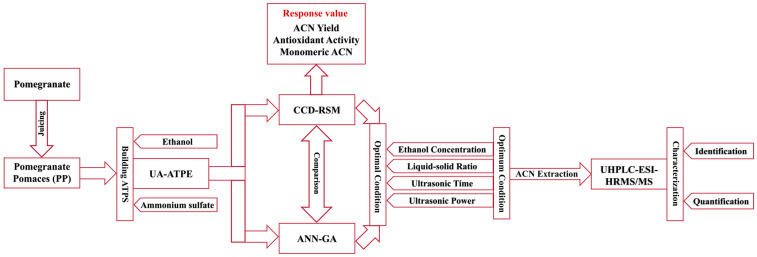
The general process roadmap for extraction, optimization, and characterization.

The provided Figure 1 illustrates a comprehensive process roadmap encompassing extraction, optimization, and characterization from PP.

### 2.2. Materials and Chemicals

The pomegranate (*Punica granatum* L.) was bought from Jianshui County, Yunnan Province, China, and refrigerated at 4 °C.

Ammonium sulfate, potassium chloride, and sodium acetate were bought from the Aladdin Reagent Co., Ltd. (Shanghai, China). Methanol, acetonitrile, and formic acid were bought from the Merck Chemical Technologies Co., Ltd. (Shanghai, China). Ethanol (AR) was bought from the Zhiyuan Chemical Reagent Co., Ltd. (Tianjin, China). Methanol (HPLC grade ≥ 99.9%) was bought from the Sigma-Aldrich Trade Co., Ltd. (Shanghai, China). Trolox (vitamin E), cyanidin-3,5-O-diglucoside (C35G), delphinidin-3-O-diglucoside (D3G), and pelargonidin-3-O-glucoside (P3G) were bought from the Shanghai Yuanye Bio-Technology Co., Ltd. (Shanghai, China). Distilled water was utilized in all experiments.

### 2.3. Preparation of Pomegranate Pomaces (PPs)

PPs were collected after pomegranates arils and were juiced by squeezing arils through a screw juicer (JYZ-E21C, Joyoung Co., Ltd., Jinan, Shandong, China), and then lyophilized at −40 ± 2 °C for 60 h in a vacuum freeze dryer (Scientz-10N, Ningbo Scientz Biotechnology Co., Ltd., Ningbo, Zhejiang, China). PP powder was obtained after grounded freeze-dried samples passed through 40 mesh sieves, and was then stored at −40 °C for subsequent use.

### 2.4. Plotting the Binodal Solubility Curve (BSC) of the Aqueous Two-Phase System (ATPS)

The ATPS was generated by mixing a specific ethanol concentration with an ammonium sulfate solution in this work. The binodal solubility curve of ethanol and ammonium sulfate was prepared by turbidimetric titration, as in the previous study [23]. Specifically, a predetermined amount of anhydrous ethanol was added dropwise to 3 mL of an ammonium sulfate solution with a concentration of 20% (*w*/*w*), and the pH value of the mixed solution was subsequently adjusted to 1.0 with hydrochloric acid. Then, a quantity of distilled water was gradually added until the cloudiness of the mixed solution disappeared completely, which was called turbidity points. Then, the solution was thoroughly shaken, and it gradually separated into two phases when left to stand. The concentrations of ethanol and ammonium sulfate were recorded at different turbidity points, which were calculated based on the sum volume of the ethanol solution, ammonium sulfate solution, and distilled water, and the BSC was plotted at 25.0 ± 0.1 °C.

### 2.5. The UA-ATPE Procedure of ACNs

For the extraction procedure of ACNs from PPs, 5 g of PP powder was mixed with a prepared ATPS (in Section 2.4) with the liquid–solid ratios of 20–60 mL/g (*v*/*w*) in a 50 mL centrifuge tube. Subsequently, the tube was closed and put into the ultrasonic bath (SB-5200DTD, Ningbo Scientz Biotechnology Co., Ltd., Ningbo, China), and the extraction temperature was fixed at 35 °C with a specific ultrasonic power and time. After ultrasonication, the solution was centrifuged at 4 °C and 8000× *g* for 10 min using a high-speed centrifuge equipped with a cooling system (Multifuge X1R, Thermo Fisher Scientific Ltd., Osterode am Harz, Germany), and then the upper liquid was collected and placed in a separatory funnel for two-phase separation. We accurately measured and recorded the volumes of the top and bottom phases when the two phases were entirely separated, with the top phase being rich in ACNs.

On the basis of pre-experiments, the factors were determined, including ethanol concentration, ultrasonic time, ultrasonic power, and the liquid–solid ratio, which had significant impacts on the ACN yield, and were used as independent variables for the UA-ATPE. According to Liu et al. [24], the ACN yield (*Y*) was calculated by dividing the content of the ACN in the top phase by the total ACN content in the PP, and the calculated formula is shown in Formula (1).

(1) $$Y=C_{t}\times \frac{V_{t}}{M_{t}}$$

where C_t_ is the concentration of the ACN in the top phase and V_t_ is the volume of the top phase. M_t_ is the sum of the ACN content of the top and bottom phases.

### 2.6. Total Monomeric ACNs

According to Lee et al.’s [25] method with slight adjustments, the total monomeric ACN concentration was measured utilizing the pH differential method. First, 3.6 mL of a potassium chloride buffer (0.025 mol/L, pH 1.0) and a sodium acetate buffer (0.4 mol/L, pH 4.5) were added to every 0.4 mL of the top and bottom phase extraction solution, respectively, and both were equilibrated in the dark for 30 min. Absorbances were determined at 510 nm and 700 nm by utilizing an ultraviolet-visible spectrophotometer (T9CS, Beijing Puxi General Instrument Co., Ltd., Beijing, China), and distilled water was used as an experimental blank. Total monomeric ACNs were calculated using Formula (2).

(2) $$Total\;monomeric\;ACNs\;content\;\left(mg/L\right)=\frac{A\times MW\times DF\times 1000}{\left(\varepsilon \times L\right)}$$

where L is the cell path length (1 cm), ε is the molar absorbance of C3G (26,900 L/mol⋅cm), DF is the dilution factor, and MW is the molecular weight of C3G (449.2 g/mol). A is calculated as [(A_510_ − A_700_) _pH1.0_ − (A_510_ − A_700_) _pH4.5_].

### 2.7. Identification and Quantification of the ACN

The ACN extraction (in Section 2.5) was analyzed by an ultra-high-performance liquid chromatography coupled to a high-resolution mass spectrometer equipped with an electrospray ionization source (Ultimate 3000, Thermo Fisher Scientific Ltd., Osterode am Harz, Germany). The separation of ACN extraction was carried out utilizing a C18 Rapid Resolution HD column (ZORBAX SB-C18, 2.1 mm × 100 mm × 1.8 µm, Agilent, Santa Clara, CA, USA) and a binary mobile phase. Eluent A was composed of 0.1% (*v*/*v*) formic acid in water, whereas eluent B had 0.1% (*v*/*v*) formic acid in acetonitrile. In negative ion scan mode, mass spectrometry was conducted with scanning under an *m*/*z* range of 100–1500. The flow rate of the mobile phase was established at 0.2 mL/min, and the sample injection volume was fixed at 2 μL through Xcalibur 4.1 software. A gradient elution was applied for 0–2 min. The mobile phase was composed of 95% eluent A. A linear gradient reduced the percentage of eluent A to 90% between 3 and 5 min. Another linear gradient decreased the proportion of eluent A to 80% between 5 and 9 min. Between 9 and 16 min, the percentage of eluent A decreased to 70%. Finally, the gradient caused the proportion of eluent A to decrease to 60% between 16 and 18 min. Between 18 and 19 min, the proportion of eluent A rose to 95% during a linear gradient, followed by a re-equilibration at 95% eluent A for 3 min. The substances were identified and quantified by comparing their mass spectrometry data to reference standard substances data, databases, or previous literature reports.

### 2.8. Central Composite Design Response Surface Model (CCD-RSM) Experimental Design

Following pre-experimentation results, CCD-RSM was used to design the experiments, and the input parameters and ranges of the five-level-four-factor were shown in Table S1. A total of 30 trials were used as independent experimental factors, including ethanol concentration (X_1_, 22–30 g/100 g), liquid-to-solid ratio (X_2_, 20–60 mL/g), ultrasonic time (X_3_, 10–50 min), and ultrasonic power (X_4_, 160–360 W). The impacts of the factors on the response scores were coded and analyzed under 30 independent experimental results. The 30 experimental settings included 16 vertical levels (+1 and −1), 8 axial levels (+α and −α), and 6 repeated intermediate levels (0). Randomization was used in the trials to reduce the impact of extraneous influences on response scores.

Design-Expert software (Version 13.0, Stat-Ease Inc., Minneapolis, MN, USA) was used to investigate the connection between experimental factors and ACN yield, as well as the statistical significance of the model. The model regression equation was obtained by calculating Formula (3).

(3) $$Y=\beta_{0}+\sum_{j=1}^{k}\beta_{j}X_{j}+\sum_{j=1}^{k}\beta_{jj}X_{j}^{2}+\sum_{i}\sum_{<j=2}^{k}\beta_{ij}X_{i}X_{j}$$

where *Y* denotes the percentage of ACN yield; *β*_0_, *β_j_*, *β_jj_*, and *β_ij_* indicate the intercept coefficient, linear, quadratic, and second-order terms’ regression coefficients, respectively; *X_i_* and *X_j_* stand for the coded variables (wherein *i* and *j* range from 1 to *k*); and *k* represents the number of independent experimental factors (*k* = 4).

To analyze the CCD-RSM model, an analysis of variance (ANOVA) was employed. The adequacy of the CCD-RSM model was assessed through the calculation of the determination coefficient (*R*^2^) and lack-of-fit, whilst the coefficient of variation (C.V.) was used to measure the relative dispersion of the experimental points.

### 2.9. Artificial Neural Network (ANN) Model Experimental Design

**Figure 2:**
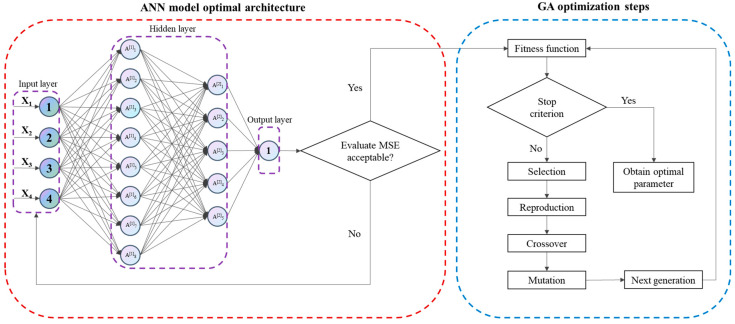
The optimal architecture of the ANN and GA optimization steps.

The target values of the ACN yield for ANN optimization and prediction were determined based on the results obtained from CCD-RSM screening. In Figure 2, the ANN structure consisted of one input layer (X_1_, X_2_, X_3_, and X_4_), two hidden layers, and one output layer (ACN yield). The ANN model was trained, and the training model was randomly partitioned into 3 subsets, including training, validating, and testing, where 11/15 (22 samples) were used for training, 2/15 (4 samples) for validating, and 2/15 (4 samples) for testing. To address the challenge posed by the limited dataset, we implemented some strategies to mitigate its impact. For example, first, we applied data normalization techniques to standardize the distributions of our training samples, thereby minimizing errors in the ANN. Second, we employed weight decay and dropout to prevent overfitting and ensure the model’s generalizability on the test set. Third, 3-fold cross-validation was employed to mitigate the limitation on the dataset. During the training process, the error between the predicted and experimental values was continuously minimized through repetition of the algorithm, until it reached the required level. The tangent sigmoid function (tansig) was used as the transfer function to construct the correlation between the hidden layer and the output layer. The error between the predicted value and the true value was calculated, and if the error did not reach the set limit of the ANN model training error, the above process was repeated. All experiment results were standardized between −1 and 1 using Equation (4). Subsequently, actual values were obtained after passing through the network’s output layer.

(4) $$M_{i}=\frac{\left(M_{max}-M_{min}\right)(N_{i}-N_{min})}{N_{max}-N_{min}}$$

where *M_i_* represents the normalized value, *M_max_* symbolizes the peak value, and *M_min_* underscores the nadir value of the scaling range. Meanwhile, *N_i_* refers to the actual data that require normalizing, with *N_max_* and *N_min_* being the peak and nadir values of the actual data.

Following the ANN modeling procedure, the customized ANN model was transformed into an efficient mathematical equation utilizing essential elements, such as weight, deviation, and transfer functions, as shown in Equations (5)–(7).

(5) $$h^{\left(1\right)}=\sigma \left(W^{\left(1\right)}X+b^{\left(1\right)}\right),X\in R^{4\times 1},W^{\left(1\right)}\in R^{8\times 4},b^{\left(1\right)}\in R^{4\times 1}$$

(6) $$h^{\left(2\right)}=\sigma \left(W^{\left(2\right)}h^{\left(1\right)}+b^{\left(2\right)}\right),W^{\left(2\right)}\in R^{5\times 8},b^{\left(2\right)}\in R^{8\times 1}$$

(7) $$\hat{y}=W^{\left(3\right)}h^{\left(2\right)}+b^{\left(3\right)},W^{\left(3\right)}\in R^{1\times 5},b^{\left(3\right)}\in R^{5\times 1}$$

### 2.10. Evaluation of the Accuracy of the Constructed Models

In order to more accurately compare and assess the predictive proficiency of the CCD-RSM and ANN models, the coefficient of determination (*R*^2^), root-mean-square error (*RMSE*), mean-square error (*MSE*), and absolute average deviation (*AAD*) were selected as the evaluation parameters [26], as shown in Equations (8)–(11):

(8) $$R^{2}=1-\frac{\sum_{i=1}^{n}{\left(x_{i}-x_{ik}\right)}^{2}}{\sum_{i=1}^{n}{\left(x_{ik}-x_{z}\right)}^{2}}$$

(9) $$MSE=\frac{1}{n}\sum_{i=1}^{n}{\left(x_{i}-x_{ik}\right)}^{2}$$

(10) $$RMSE=\sqrt{\frac{1}{n}\sum_{i=1}^{n}{\left(x_{i}-x_{ik}\right)}^{2}}$$

(11) $$AAD\left(\%\right)=\left[\frac{\sum_{i=1}^{n}\left(\left|x_{ik}-x_{i}\right|/x_{ik}\right)}{n}\right]$$

where *x_i_* represents the predicted yield, *x_ik_* is the actual or experimental yield, and *x_z_* is the average of experimental yields. Additionally, *n* denotes the number of data points and parameters within each model.

### 2.11. Optimization of the Genetic Algorithm (GA) Process

One notable advantage of the GA lies in its ability to excel in attaining comprehensive solutions rather than merely local ones [27]. In this study, a GA and a developed ANN were utilized to enhance the optimization for extracting parameters of the ACN, and the GA optimization steps as shown in Figure 2. Our ANN architecture took the form of a fully connected feedforward network, featuring two hidden layers with sizes of 8 and 5, respectively. Each of these layers was sequentially followed by a sigmoid activation function. This neural structure was adopted to convert input data comprising 4 distinct features, namely the ‘liquid-to-solid ratio’, ‘ethanol concentration’, ‘ultrasonic time’, and ‘ultrasonic power’, into a scaler output value representing ‘ACN yield’.

Our approach was initiated by training the ANN using 30 carefully curated samples to create an approximation of the functional relationship between input features and their corresponding output. These training samples were sourced from the result of UA-ATPE and were normalized prior to their input into the ANN. The *MSE* functions as our chosen loss metric, and we employed a 3-fold cross-validation procedure.

Subsequently, the GA was introduced to identify the optimal combination of input features. The GA optimization transpired across distinct stages: calculating fitness scores across the existing population, picking the most promising solutions as parent candidates within the mating pool, executing crossover and mutation operations, and iteratively repeating this progression over a predefined number of generations. The parameters were set to a population size of 1000, a maximum evolutionary generation of 20, a crossover probability uniformly distributed between [1, 3], taking random values, and a variance probability of 0.004.

The general procedure worked as follows: 30 initial populations were randomly generated using the GA, and the fitness of every individual was obtained via the developed ANN model. Genetic operation was then performed on each individual, whereby individuals of every generation used roulette to select excellent genes with large fitness values and exchanged them by employing a two-point crossover (yielding a crossover probability of [1, 3]) and random mutagenesis (yielding a mutation probability of 0.004). Subsequently, the new genotypes and assemblies were meticulously cultivated and assessed to ascertain if they complied with the algorithm’s termination criterion. Should it fail, it underwent subsequent iterations until the individual representing the maximum fitness surfaced.

### 2.12. Statistical Analysis

Statistical analysis was carried out on the data by triplicate analysis (*n* = 3). Significance was tested through ANOVA and Tukey’s multiple comparisons with a confidence level of 95% facilitated by SPSS 26.0 software (International Business Machines Corp., Almonk, NY, USA) and presented as mean ± standard deviation. Design-Expert 13.0 software was used to analyze CCD-RSM data and plot 2D or 3D graphics. The other graphics were plotted by Origin 2022 software (Origin Lab Inc., Northampton, MA, USA). Xcalibur 3.0 software (Thermo Fisher Scientific Ltd., Osterode am Harz, Germany) was used to analyze and process the UHPLC-ESI-HRMS/MS data. Python 3.11.1was used to build and operate the ANN-GA model.

## 3. Results and Discussion

### 3.1. Ethanol–Ammonium Sulphate Extraction System Phase Diagram

**Figure 3:**
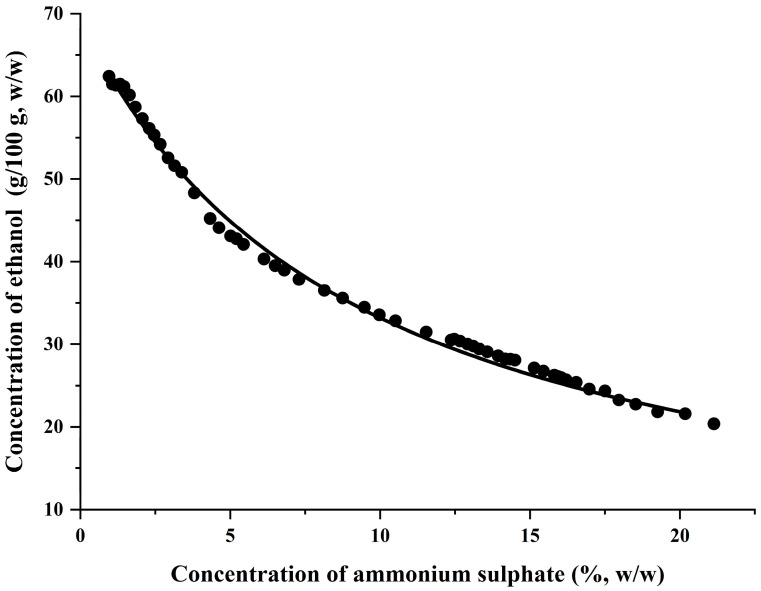
A phase diagram of ethanol and ammonium sulphate.

A suitable extraction system was essential for ACN extraction, and the ATPS phase diagram offered a comprehensive reflection on the details for liquid–liquid equilibrium, which was important reference data for building the ATPS. In the phase diagram, the above part of the BSC was the two-phase system, and the lower part was the homogeneous-phase system [28]. Based on the turbidity point data obtained experimentally, the phase diagram was plotted, as shown in Figure 3, and the area above in the binodal solubility curve (BSC) was picked for building the ATPS, which was also subsequently used in the UA-ATPE extraction of the ACN. In the ATPS, the bottom phase was a denser ammonium sulfate phase, whereas the top phase was an ethanol-rich watery phase. As expected, the ACN was, therefore, selectively transported to the top phase, while the co-existing impurities were removed to the other phase.

### 3.2. CCD-RSM Modeling

On the basis of the pre-experiment, the CCD-RSM model was utilized to refine the values of four autonomous medium factors, specifically X_1_—ethanol concentration, X_2_—liquid-to-solid ratio, X_3_—ultrasonic time, and X_4_—ultrasonic power. Eight CCD-RSM models were built based on these interactions to scrutinize the synergistic influences of these variables on ACN yield, antioxidant activity, and monomeric ACN content. The optimization results for each model are shown in Tables S2 and S3, and the *R*^2^ value of the ACN yield model is 0.9774, indicating that the model could explain 97.40% of the variability of the predicted response for ACN yield. Meanwhile, Figure S1A shows the curve of predicted values and actual values of the ACN yield model, which shows that almost all data points are on a 45° trend line, indicating that the model is highly dependable. The *R*^2^ values of the models for antioxidant activities and monomeric ACN contents were lower with values of 0.5916–0.8547, and Figure S1B–H also show that the data points of the predicted values and actual results of other models are poorly fitted to a 45° line. Thus, the ACN yield model with the highest coefficient of determination (*R*^2^ = 0.9774) was chosen for further parameter optimization and comparative analyses in Table 1. It should be noted that a high *R*^2^ value did not necessarily indicate the adequacy of the regression model because the *R*^2^ value always tends to increase with the inclusion of additional variables, even if additional variables were not statistically significant. Therefore, it is more appropriate to consider the adjusted *R*^2^ for a more accurate representation [26]. In the CCD-RSM model of ACN yield, the predicted and adjusted *R*^2^ were 0.8809 and 0.9562, respectively, and the difference between these two values was within an acceptable range of less than 0.2. The experimental results of the ACN yield model were fitted to the CCD-RSM model using multiple regression analysis, which was given in terms of a coded factor and can be found in Equation (12).

(12) $$\begin{array}{l} Y=86.20+4.26X_{1}-0.40X_{2}+4.19X_{3}+1.44X_{4}+2.77X_{1}X_{4}+1.78X_{2}X_{4}-3.22X_{3}X_{4}\\ -10.32{X_{1}}^{2}-4.98{X_{2}}^{2}-6.72{X_{3}}^{2}-5.80{X_{4}}^{2} \end{array}$$

The optimum parameters determined by the CCD-RSM model based on the equation above were an ethanol concentration of 28.6 g/100 g, a liquid-to-solid ratio of 39.8 mL/g, an ultrasonic time of 33.5 min, and an ultrasonic power of 282.9 W, with a maximum ACN production of 85.25%. As shown in Table 1, analysis of variance (ANOVA) was used to justify the ACN yield model’s significance, and the result showed that the CCD-RSM model of ACN yield was highly significant at a level of *p*-value 0.0001. Meanwhile, the coefficient of variation (C.V. = 1.11%) of the model was low, and the lack of fit was not significant (*p* = 0.0760 > 0.005), indicating that the regression equation was adequately fitted and could describe the true relationship between the factors and the response values. In general, the adequate precision represented the ratio of signal to noise, and a ratio greater than four indicated that the model results were desirable. The ACN yield model has an adequate precision value of 22.9694, indicating sufficient signals and usability for navigating the design space [26].

**Figure 4:**
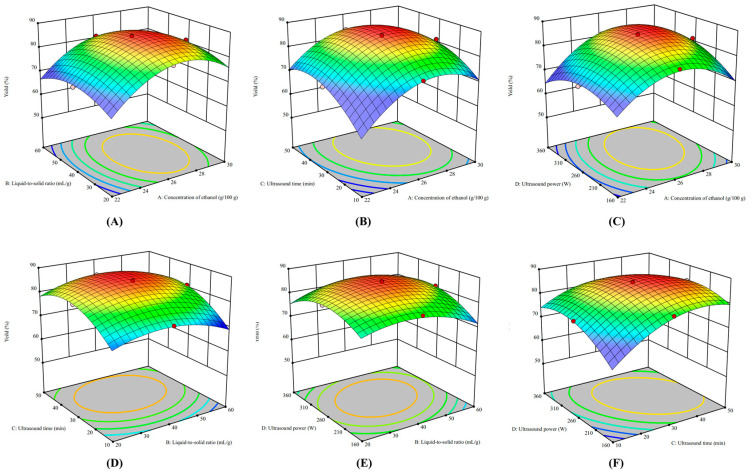
The CCD-RSM model analysis for the extraction factors on ACN yield. Factors: X_1_ (ethanol concentration) and X_2_ (liquid-to-solid ratio) and constant X_3_ (30 min) and X_4_ (260 W) (**A**). Factors: X_1_ (ethanol concentration) and X_3_ (ultrasound time) and constant X_2_ (40 mL/g) and X_4_ (260 W) (**B**). Factors: X_1_ (ethanol concentration) and X_4_ (ultrasonic power) and constant X_2_ (40 mL/g) and X_3_ (30 min) (**C**). Factors: X_2_ (liquid-to-solid ratio) and X_3_ (ultrasonic time) and constant X_1_ (26 g/100 g) and X_4_ (260 W) (**D**). Factors: X_2_ (liquid-to-solid ratio) and X_4_ (ultrasonic power) and constant X_1_ (26 g/100 g) and X_3_ (30 min) (**E**). Factors: X_3_ (ultrasonic time) and X_4_ (ultrasonic power) and constant X_1_ (26 g/100 g) and X_2_ (40 mL/g) (**F**).

The model parameters X_1_, X_3_, X_1_^2^, X_2_^2^, X_3_^2^, and X_4_^2^ were highly significant at *p* < 0.001, and X_4_, X_1_X_4,_ and X_3_X_4_ were significant at *p* < 0.05, while the other factors (X_2_, X_1_X_2_, X_1_X_3_, X_2_X_3_, X_2_X_4_) had no significant effect on ACN yield at *p* > 0.05. The above CCD-RSM model’s quadratic terms (X_1_^2^, X_2_^2^, X_3_^2^, and X_4_^2^) theoretically point to the maximum value on the response surface graph, indicating the existence of preferable parameter combinations in the four factors to maximize the ACN yield from the PP. The response surface in Figure 4 depicts the influences of the four factors on ACN yield. All of the response surface curves were convex, and the extreme point appeared on the highest of the surfaces, indicating that the mutual interaction of the four factors on ACN yield was significant.

### 3.3. Effect of Factors on ACN Yield

**Figure 5:**
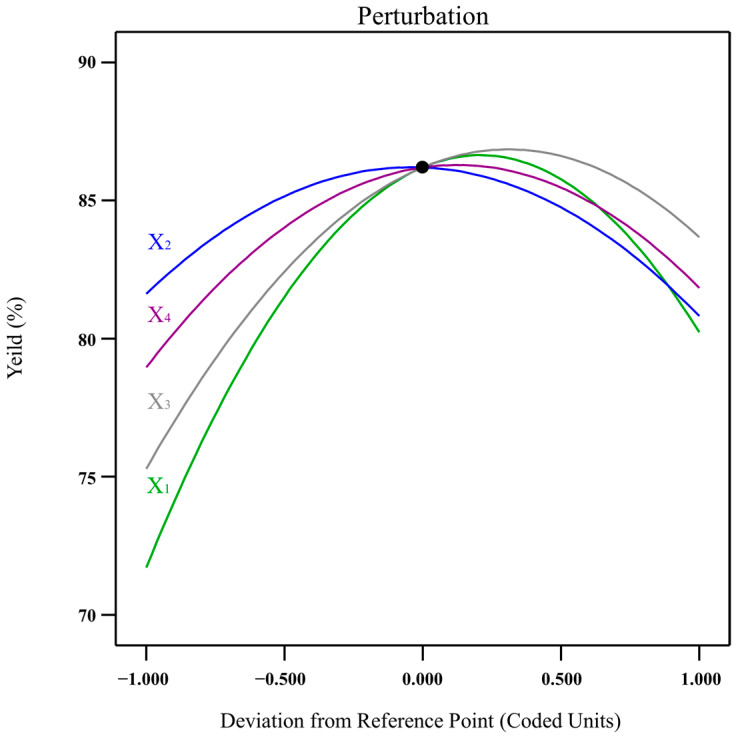
Perturbation plot of the CCD-RSM model for optimizing ACN yield from PPs. Factors: X_1_ (ethanol concentration), X_2_ (liquid-to-solid ratio), X_3_ (ultrasonic time), and X_4_ (ultrasonic power).

The individual effects of each input factor on ACN yield are depicted in Figure 5, which shows the significant effect of each factor on ACN yield. The four factors were represented by four perturbation curves, and the larger the slope of the curve, the greater the significance of the factor on ACN yield. Thus, the order of these four factors’ influences on ACN yield was ethanol concentration, ultrasonic time, ultrasonic power, and solid–liquid ratio, which also corresponded to the linear term changes in *F*-values shown in Table 1. In Figure 4A–C, the ACN yields all increased initially and decreased subsequently the ethanol concentration increased. When the ethanol concentration increased from 22 to 26 g/100 g, more ACN accumulated in the top phase as its volume increased, whereas ACN yield decreased when the ethanol concentration exceeded 26 g/100 g, which could be explained by the fact that the ethanol had a lower dielectric constant than water and it absorbed less energy under the same ultrasonic power [29]. Thus, a higher percentage of ethanol reduced the extract efficiency of UA-ATPE.

As a liquid–solid mass transfer process, the concentration gap of target components between intracellular and extracellular served as one of the propelling forces in the ATPE [30]. In this study, Figure 4A,D,E show that the total ACN yield rose as the liquid–solid ratio increased from 20 to 40 mL/g due to an increase in the liquid–solid ratio that could reduce the viscosity of the extraction medium and enhance the mass transfer driving rate between the solvent and the solids, resulting in higher ACN yields. However, when the liquid–solid ratio increased further, ACN yield steadily dropped, possibly due to the water needing to diffuse a longer distance into the interior of the PP, resulting in a limited mass transfer process. Odabaş et al. [14] found that the ACN yield extracted from *Rosa canina* fruits by MA-ATPE increased from 1071.62 to 1247.73 mg c3ge/100 g when the liquid–solid ratio increased from 20 to 40 mL/g, and then ACN yield showed no significant change when the liquid–solid ratio increased constantly. A similar result was reported for the extraction of polysaccharides from lilies by utilizing UA-ATPE [11].

As shown in Figure 4B,D,F, ACN yield was significantly enhanced when the ultrasonic time was prolonged from 10 to 30 min, but ACN yield decreased sharply after 30 min. The above results showed that the ultrasonic time had a substantial influence on ACN yield, as cavitation and mechanical effects during ultrasonic processing may cause disruptions to the cell wall, leading to a surge in the surface contact area between the solid and liquid, resulting in faster solvent penetration into the plant matrix, thereby boosting mass transfer and favoring greater extraction rates [31]. Figure 4C,E,F show that increasing ultrasonic power from 160 to 260 W could increase ACN yield. However, when the ultrasonic power was raised from 310 to 360 W, the ACN yield gradually decreased, corresponding with the results of previous reports [32]. The decreases in ACN yield with higher ultrasonic power and longer time were partly because the cavitation bubbles formed became unprecedentedly high when the ultrasonic time or power increased to a certain degree, which enhanced energy dissipation within the cavitation bubbles and promoted inadequate collapse, thereby reducing energy transfer efficiency and, ultimately, ACN yield [33,34,35]. Furthermore, an increase in heat and the production of hydroxyl radicals caused by ultrasonic treatment could lead to the opening of anthocyanin rings and the formation of chalcone, resulting in the degradation of ACNs [36]. A similar result was found by Lou et al. [37], who found that the yields of chickpea oil significantly improved from 59.71 to 83.32% when the ultrasonic power was elevated from 100 to 250 W with an extraction time of 20 min, and the chickpea oil yield slightly decreased when the ultrasonic power was more than 250 W.

In Figure 4 and Table 1, the interaction of several parameters on ACN yield is depicted. The interaction term (X_3_X_4_) between the ultrasonic time (X_3_) and ultrasonic power (X_4_) had a *p*-value of 0.0026, indicating that its interaction on ACN yield was the most significant. This might be because ultrasonication was a key factor in promoting the release of ACNs from PPs, and the time and power are two main measurement parameters of ultrasonic energy. The appropriate ultrasonic energy could promote the release and extraction of ACNs, but when the ultrasonic energy exceeds a certain threshold, it will lead to the generation of hydroxyl radicals and thermal effects, which cause ACN degradation, thus directly reducing ACN yield [38]. The interaction term (X_1_X_4_) also had a lower *p*-value of 0.0072, which indicated that the interaction between ethanol concentration (X_1_) and ultrasonic power (X_4_) also significantly affected ACN yield. Among the interaction term (X_1_X_4_), the influence of ethanol concentration was stronger than the ultrasonic power on ACN yield since ACN yields increased from 67 to 80% when the ethanol concentration increased from 22 to 26 g/100 g, which was significantly greater than the increased value after increasing ultrasonic power.

### 3.4. ANN Modeling

**Figure 6:**
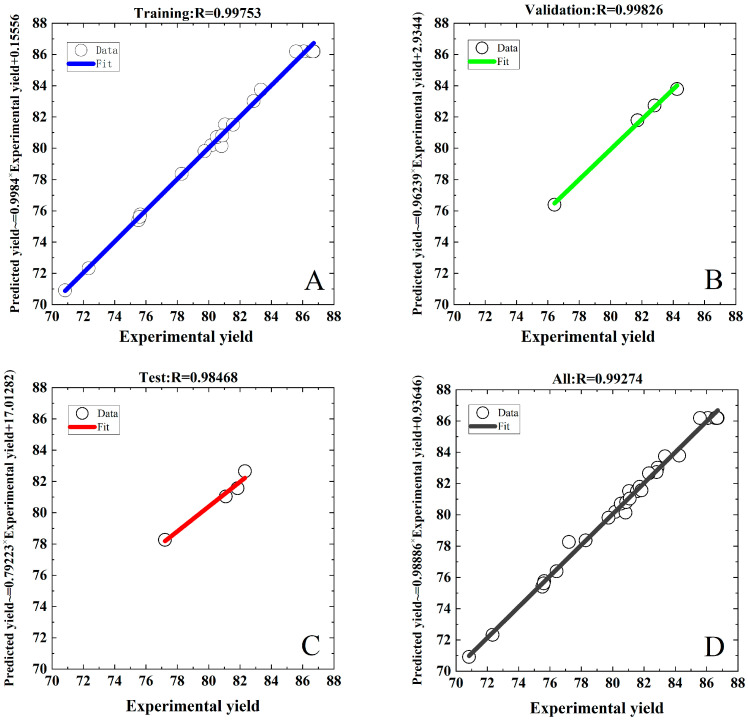
Graphical representation of the results showing a comparison between experimental and predicted yield values by the ANN. Training (22 runs) (**A**), validation (4 runs) (**B**), testing (4 runs) (**C**), and overall (30 runs) (**D**).

The target output index of the ANN model was determined as the ACN yield with the highest *R*^2^ value, which was obtained through optimization and prediction using the CCD-RSM model. The ANN was used to develop a mathematical model that determined the relationship between ACN yield and the process parameters. Figure 6 depicts a regression diagram of the experimental data with the computed ANN prediction data for training, testing, validation, and all datasets. The data were first normalized through the standardization process to reduce the errors of the neural network parameters and response variables, and the optimal ANN model was used to predict ACN yield from the experimental variables. The *R*-values for the training and validation datasets were 0.9975 and 0.9983, respectively, indicating a good correlation between the experimental data utilized to build the model and the predicted data. The *R*-value for the test dataset was observed to be 0.9847, reflecting the good predictive accuracy of the ANN model for unanticipated data. Furthermore, there was an *R*-value of 0.9927 for all datasets, which exhibited that 99% of the model-predicted and experimental data were in close agreement, and only 1% of the variance was not explained by the ANN model. The results demonstrated that the ANN model based on the test data had excellent predictive power and high accuracy. Muthusamy et al. [39] optimized the extraction process of pectin from *Helianthus annuus* (sunflower) heads by the ANN model, and the *R*-values for training, validation, and testing dataset were 0.9983, 0.9995, and 0.9999, respectively, which indicated that the ANN model was suitable for the prediction and optimization of extracted pectin.

### 3.5. ANN-GA Optimization

**Figure 7:**
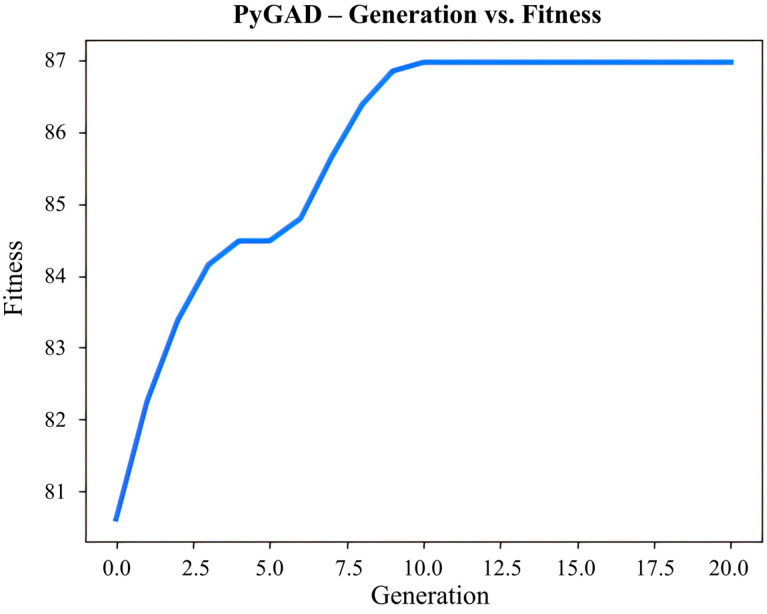
The fitness curve of the GA optimization process.

The GA was utilized to optimize the process conditions to achieve a higher ACN yield from PPs based on the ANN model. Figure 7 displays the fitness curve of the relationship between the number of iterations and ACN yield, which was obtained by ANN-GA optimization. After 10 iterations, the optimal fitness was obtained, and the ACN yields reached the maximum. The optimized conditions proposed by the ANN-GA were an ethanol concentration of 28.3 g/100 g, a liquid-to-solid ratio of 49.0 mL/g, an ultrasonic time of 27.4 min, and an ultrasonic power of 330.4 W, with a maximum ACN yield of 86.98%. To make parameter control easier during the experiment, the above extraction parameters were practically modified as follows: an ultrasonic power of 28%, a liquid-to-solid ratio of 49.0 mL/g, an ultrasonic time of 27 min, and an ultrasonic power of 330 W. According to the above parameters, the experimental value of ACN yield was 87.82%. The overall result indicated that the measured and predicted values of ACN yield agreed with a 95% confidence interval. In essence, the ANN-GA methodology worked admirably to improve ACN yield from PPs, and the model was able to accurately represent the internal correlation between ACN yield and experimental variables with superior simulation performance. In addition, based on the ANN principle, it also suggested that the ACN extraction process was not a linear biosystem. Thus, the deviations between experimental and predicted values of an integrated ANN-GA model were less and defined a better fitness of the predicted model.

### 3.6. Evaluation and Comparison of the ANN-GA and CCD-RSM Models

The experimental results and predicted values of the ANN-GA and CCD-RSM models are represented in Table S2. By plotting the experimental values and predicted results of the two models (ANN-GA and CCD-RSM), results showed that both models were well-designed and produced a perfect match, and the ANN-GA had an accurate predictive capacity compared to CCD-RSM, with a higher *R*^2^ value (0.9940 compared to 0.9773) and a lower *MSE* (0.0056 compared to 0.0083), *RMSE* (0.4322 compared to 0.6311), and *AAD* (0.2922% compared to 0.6211%). As shown in Table 2, the *R*^2^ values suggested that the independent variables account for 97.73% and 99.40% of the variations in the respective ACN yields by the CCD-RSM and ANN-GA models. The mean squared error (*MSE*) was a measure of the accuracy of a model’s predictions. The lower the *MSE*, the better the model’s predictions. The *MSE* values for the training and verification samples were 0.0083 and 0.0056 for the ANN-GA models, respectively, indicating that there was a good correlation between the predicted values and samples.

**Figure 8:**
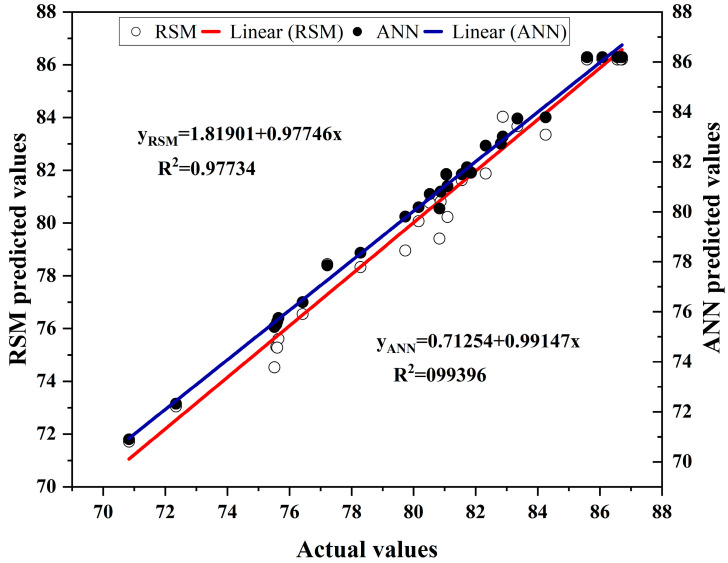
Relationship between the actual and predicted values using the CCD-RSM and ANN-GA models.

Furthermore, the experimental values were compared with the predicted values of ACN yield using the CCD-RSM model and the ANN-GA model, with the results displayed in Figure 8. It could be shown that the ANN-GA model has a higher fitting degree, precision, and prediction ability than the CCD-RSM model. Muthusamy et al. [39] found that the ANN-GA model (*R*^2^ = 0.99) showed a better performance in the optimization of extraction parameters for pectin from sunflower heads than the RSM model (*R*^2^ = 0.96), and the predicted extraction rates were more precise [10]. Zhang et al. [11] compared the optimization of polysaccharides and polyphenols extracted from *Schisandra chinensis* by the RSM and ANN-GA, and the ANN-GA model exhibited higher *R*^2^ values (0.9924 compared to 0.9774) and lower values of *χ*^2^ (0.0642 compared to 0.2119), *AAD* (0.9022 compared to 1.7713), and *RMSE* (0.1820 compared to 0.3143) than the RSM model. However, a different result was found by Karmakar et al. [40], who found that the RSM and ANN models were compared to optimize the extraction parameters of starch from the corms of *A. paeoniifolius*, and the *R*^2^ values of the RSM and ANN were 0.999 and 0.602, respectively, which indicated that the RSM model performed better than the ANN model. Jha et al. [41] compared the optimization of RSM coupled with desirability function (RSM-DF), RSM coupled with a genetic algorithm (RSM-GA), and the ANN-GA model for supercritical fluid extraction of phytochemicals from *Terminalia chebula* pulp, and the results showed RSM models were better than the ANN model for predicting the responses. The differences in performance in the predicted results of the ANN-GA and CCD-RSM models applied for different extraction processes could be mainly attributed to the fact that the extraction process for different target substances in the extraction system was different than the selection of parameters for constructing models.

### 3.7. Characterization and Evaluation of ACN Extraction

**Figure 9:**
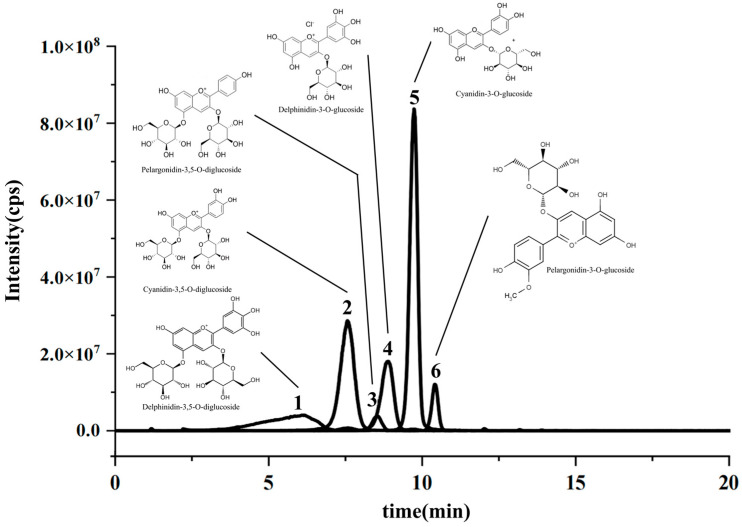
The total ion chromatogram of the ACN extraction from the PP.

The pomegranate contains three main types of ACNs: delphinidin, cyanidin, and pelargonidin, which bond with sugar to form glycosidic compounds. The most common ACNs found in pomegranates include pelargonidin-3-diglucoside, delphinidin-3,5-diglucoside, cyanidin-3-glucoside, delphinidin-3-glucoside, delphinidin-3,5-diglucoside, and so on [42]. As shown in Figure 9, a total of six ACNs from PPs were identified based on the peak time and ion fragment information (MS, MS^2^). As shown in Table 3, the content of cyanidin-3-O-glucoside (peak 5) was the highest, reaching 57.01 ± 1.36 mg/g DW, followed by cyanidin-3,5-O-diglucoside (peak 2) and delphinidin-3-O-glucoside (peak 4) with values of 31.35 ± 0.58 and 18.23 ± 0.22 mg/g DW, respectively. The main ACN in the extraction ACNs from PPs was consistent with a previous study [43,44,45].

## 4. Conclusions

In this study, both the CCD-RSM and ANN-GA models could accurately predict the ACN yield from PPs, and the ANN-GA model predicted ACN yield better and had a higher degree of fit and accuracy, as well as a better pre-estimation ability. The model built with the ANN-GA was experimentally validated, and the process parameters were as follows: an ethanol concentration of 28 g/100 g, a liquid-to-solid ratio of 49.0 mL/g, an ultrasonic time of 27 min, and an ultrasonic power of 330 W. A maximum value of 86.98% was anticipated by the above model, and the experimental maximum value was 87.82%, which was within the 95% confidence interval. Overall, the ANN-GA model better fitted the internal link between ACN yield and experimental parameters with excellent simulation performance, and there was an excellent degree of agreement between the predicted and actual results. A total of six ACNs were identified in the extracted ACN, among which the highest content of cyanidin-3-O-glucoside reached 57.01 ± 1.36 mg/g DW. Thus, this study succeeded in building an efficient and green way to extract ACNs from PPs by optimizing and forecasting the extraction process using the ANN-GA model based on the UA-ATPE method, which provided a novel idea and methodology for the high-value utilization of abundant PP resources produced with the development of the pomegranate industry. This study also offered a foundation for the extraction of bioactive substances from by-products of fruit and vegetable processing, which has positive significance for exploring the potential value of more by-products and obtaining good ecological and economic benefits in the future.

## Figures and Tables

**Table 1:** ANOVA of the CCD-RSM model for the ACN yields based on the UA-ATPE method.

Source	Sum of Squares	*Df* ^a^	Mean Square	*F*-Value	*p*-Value
Linear					
X_1_	108.89	1	108.89	136.79	<0.0001
X_2_	0.9600	1	0.9600	1.21	0.2894
X_3_	105.50	1	105.50	132.54	<0.0001
X_4_	12.47	1	12.47	15.67	0.0013
Interactions					
X_1_X_2_	0.1640	1	0.1640	0.2061	0.6564
X_1_X_3_	2.74	1	2.74	3.44	0.0834
X_1_X_4_	7.67	1	7.67	9.64	0.0072
X_2_X_3_	0.6162	1	0.6162	0.7741	0.3928
X_2_X_4_	3.17	1	3.17	3.98	0.0645
X_3_X_4_	10.37	1	10.37	13.03	0.0026
Quadratic					
X_1_^2^	179.43	1	179.43	225.42	<0.0001
X_2_^2^	42.53	1	42.53	53.43	<0.0001
X_3_^2^	77.43	1	77.43	97.28	<0.0001
X_4_^2^	77.43	1	57.69	72.47	<0.0001
Lack of Fit	10.56	10	1.06	3.82	0.0760
Pure Error	1.38	5	0.2763		
Residue	11.94	1			
Model	515.32	14	36.81	46.24	<0.0001
Total SS	527.26	29			
*R* ^2^	0.9774				
Adjusted *R*^2^	0.9562				
Predicted *R*^2^	0.8809				
Adeq Precision	22.9694				

All data were acquired from three repeated experiments: X_1_—ethanol concentration (*w*/*w*, g/100 g); X_2_—liquid-to-soild ratio (*v*/*w*, mL/g); X_3_—ultrasouic time (min); X_4_—ultrasonic power (W); *Df* ^a^—degree of freedom.

**Table 2:** Calculated predictive capacity of the CCD-RSM and ANN-GA models for ACN yield.

Calculated Predictive Capacity	CCD-RSM	ANN-GA
*R* ^2^	0.9773	0.9940
*MSE*	0.0083	0.0056
*RMSE*	0.6311	0.4322
*AAD* (%)	0.6211	0.2922

All data were acquired from three repeated experiments: *R*^2^: coefficient of determination; *MSE*: mean squared error; *RMSE*: root-mean-square error; *AAD*: absolute average deviation.

**Table 3:** Molecular structure identification of ACN extractions from PPs by UHPLC-ESI-HRMS/MS.

Peak No.	RT (min)	Anthocyanin	Molecular Formula	MS[M]^+^ (*m*/*z*)	MS^2^	Content (mg/g DW)
1	1.37	Delphinidin-3,5-O-diglucoside	C_27_H_31_O_17_	627.1555	303.0501, 304.0534, 465.1028	8.79 ± 1.54
2	7.61	Cyanidin-3,5-O-diglucoside	C_27_H_31_O_16_	611.1606	271.0596	31.35 ± 0.58
3	9.05	Pelargonidin-3,5-O-diglucoside	C_27_H_31_O_15_	595.1657	287.0551, 449.1081	3.33 ± 0.12
4	9.28	Delphinidin-3-O-glucoside	C_27_H_31_O_12_	465.1028	303.0497	18.23 ± 0.22
5	10.05	Cyanidin-3-O-glucoside	C_21_H_21_O_11_	449.1078	287.0549, 449.1090	57.01 ± 1.36
6	10.46	Pelargonidin-3-O-glucoside	C_21_H_31_O_10_	433.1127	271.0603, 433.1134	7.30 ± 0.39

All data were acquired from three repeated experiments. The content data represented are the means ± standard deviation.

## Data Availability

All data are contained within the article or Supplementary Materials.

## Supplementary Materials

The following supporting information can be downloaded at www.mdpi.com/xxx/s1, Figure S1: The predicted response value and actual experimental value of ACN extraction from PP were optimized by the CCD-RSM model; Table S1: The coded and actual levels of each factors used for CCD-RSM model; Table S2: The CCD-RSM experimental design and results for ACN yield including actual and predicted yields by models, antioxidant activity and monomeric ACNs; Table S3: The fit summary of all the response value for ACN extraction based on CCD-RSM model. Antioxidant activity determination method in Supplementary Materials. References [46,47,48,49] are cited in the Supplementary Materials.
