# Supplementary material for: Comparison of an Ultrasound-Assisted Aqueous Two-Phase System Extraction of Anthocyanins from Pomegranate Pomaces by Utilizing the Artificial Neural Network–Genetic Algorithm and Response Surface Methodology Models
Source: Foods. 2024 Jan 8;13(2):0. doi: 10.3390/foods13020199 (PMC11154380; doi:10.3390/foods13020199)
Supplement: Supplementary file 1 [file foods-13-00199-s001.zip › foods-2783106-supplementary.pdf]

Table S1

The coded and actual levels of each factors used for CCD-RSM model.

LEVEL	X_1-Ethanol concentration (w/w, g/100 g)	X_2-Liquid-to-soild ratio (v/w, mL/g)	X_3-Ultrasounic time (min)	X_4-Ultrasounic power (W)
-α	22	20	10	160
-1	24	30	20	210
0	26	40	30	260
+1	28	50	40	310
+α	30	60	50	360

Table S2

The CCD-RSM experimental design and results for ACN yield including actual and predicted yields by models, antioxidant activity and monomeric ACNs.

A	X_1	X_2	X_3	X_4	Y_1	Y_2	Y_3	Y_4	Y_5	Y_6	Y_7	Y_8	Y_9	Y_{10}
1	22	40	30	260	70.83	71.71	70.90	154.67	215.73	153.69	1.4713	0.0097	0.0017	0.0040
2	24	30	20	310	75.57	75.31	75.50	207.17	243.11	210.35	1.5650	0.0102	0.0018	0.0048
3	24	50	40	210	80.16	80.07	80.19	170.64	224.06	168.07	1.5171	0.0106	0.0018	0.0043
4	24	30	40	310	78.28	78.33	78.37	224.94	240.13	294.26	1.5897	0.0100	0.0020	0.0045
5	24	50	40	310	80.83	79.41	80.14	205.50	235.01	233.78	1.5535	0.0099	0.0023	0.0045
6	24	30	40	210	80.52	80.77	80.72	183.83	233.82	178.07	1.5244	0.0106	0.0194	0.0042
7	24	50	20	210	72.34	73.05	72.33	177.86	227.27	164.16	1.5631	0.0120	0.0022	0.0036
8	24	50	20	310	75.64	75.61	75.76	197.03	241.32	215.88	1.5942	0.0100	0.0019	0.0047
9	24	30	20	210	75.51	74.53	75.40	196.33	217.63	181.40	1.5093	0.0102	0.0025	0.0038
10	26	40	30	360	81.05	81.84	81.52	196.61	243.46	215.59	1.6424	0.0098	0.0020	0.0048
11	26	40	30	260	85.59	86.20	86.19	193.97	241.68	239.21	1.6241	0.0105	0.0019	0.0045
12	26	40	30	260	86.71	86.20	86.19	150.50	228.11	145.69	1.5783	0.0090	0.0018	0.0036
13	26	60	30	260	80.87	80.82	80.82	205.92	244.77	215.02	1.6245	0.0108	0.0021	0.0040
14	26	20	30	260	81.55	81.62	81.51	204.39	243.35	247.40	1.6553	0.0106	0.0023	0.0039
15	26	40	30	260	86.08	86.20	86.19	201.33	244.42	208.16	1.6177	0.0094	0.0018	0.0046
16	26	40	10	260	75.60	75.28	75.61	152.44	224.65	130.45	1.5571	0.0095	0.0022	0.0068
17	26	40	30	260	85.58	86.20	86.19	149.67	224.54	138.64	1.5848	0.0093	0.0019	0.0055
18	26	40	30	160	79.73	78.96	79.81	187.31	240.61	189.11	1.6103	0.0093	0.0018	0.0047
19	26	40	30	260	86.56	86.20	86.19	230.36	244.30	267.21	1.6148	0.0092	0.0022	0.0050
20	26	40	50	260	83.34	83.67	83.74	187.72	242.27	210.35	1.6460	0.0100	0.0019	0.0046
21	26	40	30	260	86.67	86.20	86.19	234.53	244.77	330.16	1.7681	0.0114	0.0021	0.0053
22	28	50	40	310	82.87	84.03	83.01	225.92	244.77	232.83	1.7385	0.0097	0.0022	0.0046
23	28	30	40	210	82.81	83.02	82.74	227.72	244.89	249.69	1.6860	0.0094	0.0020	0.0051
24	28	30	20	310	81.72	81.98	81.79	219.39	238.46	218.16	1.6057	0.0097	0.0026	0.0061
25	28	30	40	310	84.25	83.35	83.79	203.42	244.42	221.69	1.6073	0.0099	0.0019	0.0048
26	28	50	20	210	76.42	76.55	76.39	232.44	244.65	312.73	1.7110	0.0106	0.0021	0.0060
27	28	50	40	210	81.85	81.92	81.57	229.11	244.89	258.54	1.6912	0.0102	0.0023	0.0048
28	28	30	20	210	77.21	78.44	78.26	229.94	244.89	319.40	1.6893	0.0097	0.0019	0.0049
29	28	50	20	310	82.32	81.88	82.66	232.17	244.89	254.16	1.6425	0.0096	0.0018	0.0046
30	30	40	30	260	81.09	80.23	81.03	210.36	243.11	220.92	1.5914	0.0087	0.0017	0.0046

All data was acquired from three repeated experiments; A-Run Order. X_1-Concentration of ethanol (w/w, g/100 g); X_2-Liquid-to-soild ratio(v/w, mL/g); X_3-Ultrasounic time (min); X_4-Ultrasounic power (W); Y_1-Yields Experimental(%); Y_2-Yields Predicted for RSM(%); Y_3-Yields Predicted for ANN(%); Y_4- DPPH (mmol/L); Y_5-ABTS (mmol/L); Y_6-FRAP (mmol/L); Y_7-Cyanidin-3-glucoside (mg/L); Y_8-Cyanidin-3,5-O-diglucoside(mg/L); Y_9-Pelargonidin-3-O-glucoside(mg/L); Y_{10}-Delphinidin-3-O-diglucoside (mg/L).

Table S3

The fit summary of all the response value for ACN extraction based on CCD-RSM model.

Source	ACNs Yield	DPPH	ABTS	FRAP	C3G	C35G	P3G	D3G
P-value	< 0.0001	0.2041	0.0043	0.0336	0.0005	0.0005	0.0060	0.0006
P_{lof}-value	0.1291	0.8439	0.9950	0.8250	0.4474	0.3047	0.4995	0.2088
F-value	46.24	1.55	4.26	2.69	6.30	6.27	3.96	6.23
F_{lof}-value	3.82	0.4818	0.1401	0.5103	1.15	1.55	1.04	1.98
R^2	0.9774	0.5916	0.7990	0.7152	0.8547	0.8541	0.7873	0.8533
Adjusted R^2	0.9526	0.2104	0.6114	0.4491	0.7190	0.7180	0.5887	0.7164

All data was acquired from three repeated experiments; C3G: Cyanidin-3-glucoside; C35G: Cyanidin-3,5-O-diglucoside; P3G: Pelargonidin-3-O-glucoside; D3G: Delphinidin-3-O-diglucoside.

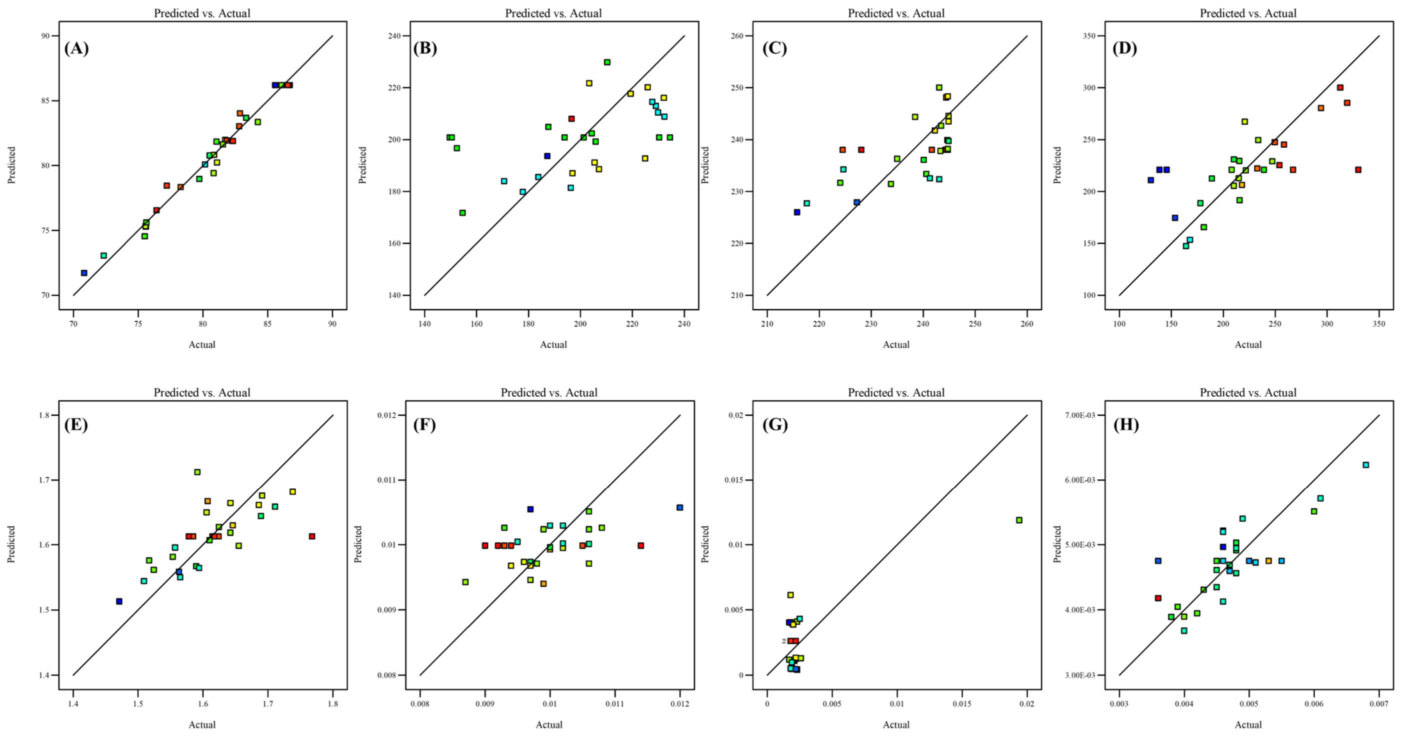

Figure. S1. The predicted response value and actual experimental value of ACN extraction from PP were optimized by the CCD-RSM model: ACNs yield (A), DPPH (B), ABTS (C), FRAP (D), Cyanidin-3-glucoside (E), Cyanidin-3,5-O-diglucoside (F), Pelargonidin-3-O-glucoside (G), Delphinidin-3-O-diglucoside (H).

Determination of antioxidant activity

Firstly, the top phase rich in ACNs was lyophilized in a vacuum freeze dryer (Scientz-10N, Ningbo Scientz Biotechnology Co., Ltd, China), and then 0.1 g of the lyophilized ACNs extraction was subsequently dissolved in 10 mL of 80% methanol, centrifuged at 6000×g for 5 min at 4 ℃ (Multifuge X1 R high-speed freezing centrifuge, Thermo Fisher Scientific Ltd., Germany). The supernatant as the ACNs extraction was used to determine antioxidant capacity of DPPH, ABTS and FRAP radical scavenging capacity.

(1)DPPH assay

DPPH radical scavenging activity was determined by a method previously reported by Sun et al.[1], with minor modifications. The working DPPH solution was obtained by diluting 0.2 mM DPPH with 80% methanol to get an absorbance value of 0.90 ± (0.05) at 517 nm. Then, 40 μL of ACNs extraction and 160 μL of working DPPH solution were completely mixed and incubated at room temperature away from light for 30 min. The absorbances of mixture was measured at 517 nm with a microplate reader (EPOCH2, BioTek Instrument Co., Ltd., USA). A calibration curve was measured with Trolox in the concentrations range of 0–1000 μmol/L. The results were expressed as mmol Trolox equivalent (TE)/g DW of ACNs extraction.

(2)ABTS assay

ABTS radical scavenging activity was determined as described by He et al. [2]. ABTS radical was produced by reacting 7 mM ABTS stock solution with 2.45 mM potassium persulfate and kept in the dark at room temperature for 12–16 h before use. The ABTS working solution was obtained by diluting with 80% methanol to get an absorbance value of 0.70 (±0.02) at 734 nm. Then, 40 μL of ACNs extraction and 160 μL of ABTS working solution were completely mixed and incubated at room temperature away from light for 6 min. The absorbances of mixture at 734 nm were measured with a microplate reader (EPOCH2, BioTek Instrument Co., Ltd., USA). A calibration curve was measured with Trolox in the concentrations range of 0–300 μmol/L. The results were expressed as mmol Trolox equivalent (TE) /g DW of ACNs extraction.

(3)FRAP assay

FRAP assay described by Benzie & Strain [3] and Buchweitz et al. [4] were used for the determination. In brief, a working solution comprising 25 mL of 0.3 M acetate buffer (pH 3.6), 2.5 mL of 10 mM TPTZ solution, and 2.5 mL of 20 mM ferric chloride solution were mixed and incubated for 1 h at room temperature. Then, 0.1 mL of ACNs extraction was mixed with 2.4 mL of working solution for 10 min at room temperature in the dark. The absorbances at 593 nm were measured with a microplate reader (EPOCH2, BioTek Instrument Co., Ltd., USA). A calibration curve was measured with Trolox in the concentrations range of 0–1000 μmol/L. The results were expressed as mmol Trolox equivalent (TE)/g DW of ACNs extraction.

References

1. Sun, D.; Huang, S.; Cai, S.; Cao, J.; Han, P. Digestion property and synergistic effect on biological activity of purple rice (Oryza sativa L.) anthocyanins subjected to a simulated gastrointestinal digestion in vitro. *Food Research International.* **2015**, *78*, 114-123, https://doi.org/10.1016/j.foodres.2015.10.029.
2. He, Z.; Tao, Y.; Zeng, M.; Zhang, S.; Tao, G.; Qin, F.; Chen, J. High pressure homogenization processing, thermal treatment and milk matrix affect in vitro bioaccessibility of phenolics in apple, grape and orange juice to different extents. *Food Chemistry.* **2016**, *200*, 107-116, https://doi.org/10.1016/j.foodchem.2016.01.045.
3. Benzie, I.F.; Szeto, Y. Total antioxidant capacity of teas by the ferric reducing/antioxidant power assay. *Journal of Agricultural and Food Chemistry.* **1999**, *47*, 633-636, https://doi.org/10.1021/jf9807768.

4. Buchweitz, M.; Speth, M.; Kammerer, D.; Carle, R. Impact of pectin type on the storage stability of black currant (*Ribes nigrum L.*) anthocyanins in pectic model solutions. *Food Chemistry*. **2013**, *139*, 1168-1178, https://doi.org/10.1016/j.foodchem.2013.02.005.
